# MCAH-ACO: A Multi-Criteria Adaptive Hybrid Ant Colony Optimization for Last-Mile Delivery Vehicle Routing

**DOI:** 10.3390/s26020401

**Published:** 2026-01-08

**Authors:** De-Tian Chu, Xin-Yu Cheng, Lin-Yuan Bai, Hai-Feng Ling

**Affiliations:** Field Engineering College, Army Engineering University of PLA, Nanjing 210007, China; detian_chu@aeu.edu.cn (D.-T.C.); cxy1999@aeu.edu.cn (X.-Y.C.); linyuan_bai@aeu.edu.cn (L.-Y.B.)

**Keywords:** multi-criteria optimization, vehicle routing problem, ant colony optimization, adaptive hybrid algorithm, last-mile delivery

## Abstract

The growing demand for efficient last-mile delivery has made routing optimization a critical challenge for logistics providers. Traditional vehicle routing models typically minimize a single criterion, such as travel distance or time, without considering broader social and environmental impacts. This paper proposes a novel Multi-Criteria Adaptive Hybrid Ant Colony Optimization (MCAH-ACO) algorithm for solving the delivery vehicle routing problem formulated as a Multiple Traveling Salesman Problem (MTSP). The proposed MCAH-ACO introduces three key innovations: a multi-criteria pheromone decomposition strategy that maintains separate pheromone matrices for each optimization objective, an adaptive weight balancing mechanism that dynamically adjusts criterion weights to prevent dominance by any single objective, and a 2-opt local search enhancement integrated with elite archive diversity preservation. A comprehensive cost function is designed to integrate four categories of factors: distance, time, social-environmental impact, and safety. Extensive experiments on real-world data from the Greater Toronto Area demonstrate that MCAH-ACO significantly outperforms existing approaches including Genetic Algorithm (GA), Adaptive GA, and standard Max–Min Ant System (MMAS), achieving 12.3% lower total cost and 18.7% fewer safety-critical events compared with the best baseline while maintaining computational efficiency.

## 1. Introduction

The COVID-19 pandemic has fundamentally reshaped consumer purchasing behavior, accelerating the shift from in-store to online platforms. In Canada, retail e-commerce sales nearly doubled within three months after the onset of the 2020 pandemic, driving unprecedented demand for fast, convenient, and reliable parcel delivery [1]. Last-mile delivery—the final leg of parcel transport to customer households—is widely recognized as the most critical yet expensive and least efficient component of logistics operations [2,3].

Many large e-commerce and logistics companies continue to employ routing strategies based solely on minimizing travel time or distance [4]. However, the rapidly growing fleet of delivery vehicles contributes significantly to urban congestion and environmental pollution [5]. Consequently, multi-criteria routing strategies that integrate environmental sustainability and safety factors have become essential for responsible logistics planning. This aligns with emerging safety-first planning frameworks that escalate verification under uncertainty to improve robust decision-making in high-risk navigation scenarios [6].

Despite considerable progress in applying meta-heuristic algorithms such as Genetic Algorithms (GA) and Ant Colony Optimization (ACO) to vehicle routing problems [7], existing approaches suffer from several key limitations. Most methods typically optimize a single aggregated objective, failing to balance trade-offs among competing criteria. Furthermore, standard pheromone update mechanisms in ACO may cause premature convergence toward locally optimal but globally suboptimal solutions. The lack of local search refinement also limits solution quality in complex multi-constraint scenarios.

To address these challenges, this paper proposes a novel Multi-Criteria Adaptive Hybrid Ant Colony Optimization (MCAH-ACO) algorithm for solving the delivery vehicle routing problem formulated as a Multiple Traveling Salesman Problem (MTSP). The proposed algorithm introduces three main contributions. First, we develop a multi-criteria pheromone decomposition strategy that maintains separate pheromone matrices for distance, time, social-environmental, and safety objectives, enabling balanced optimization across all criteria. Second, we propose an adaptive weight balancing mechanism that dynamically adjusts criterion weights based on convergence feedback, preventing any single objective from dominating the search. Third, we integrate a 2-opt local search enhancement with an elite archive that preserves solution diversity while accelerating convergence toward high-quality solutions.

Extensive experiments on real-world delivery data from the Greater Toronto Area demonstrate that MCAH-ACO achieves significant improvements over existing baselines, reducing total routing cost by 12.3% and safety-critical events by 18.7% compared with the best-performing baseline algorithm.

## 2. Related Work

### 2.1. Vehicle Routing and MTSP Optimization

The Vehicle Routing Problem (VRP) and its multi-salesman generalization (MTSP) have been extensively studied in operations research and logistics optimization. VRP typically aims to determine optimal routes for a fleet of vehicles to serve a set of customers, with common objectives including minimizing total travel distance, minimizing travel time, minimizing operational costs, maximizing vehicle utilization, and improving customer satisfaction. Key factors considered in VRP formulations include time windows, vehicle capacity constraints, heterogeneous fleets, and dynamic customer requests.

Optimization methods for VRP can be broadly categorized into three classes: (1) exact methods such as branch-and-bound and branch-and-cut, which guarantee optimal solutions but are computationally prohibitive for large instances; (2) classical heuristics including nearest neighbor, savings algorithm, and sweep algorithm, which provide fast but often suboptimal solutions and (3) meta-heuristics such as Genetic Algorithms (GA), Ant Colony Optimization (ACO), Simulated Annealing (SA), and Particle Swarm Optimization (PSO), which offer good trade-offs between solution quality and computational efficiency.

Meta-heuristic approaches, particularly GA and ACO, remain dominant due to their robustness and scalability for large-scale instances, as comprehensively surveyed by Cheikhrouhou and Khoufi [8]. The Max-Min Ant System (MMAS) [9] introduced pheromone bounds to prevent stagnation and remains a strong baseline. Chen et al. [4] model VRP as MTSP and reduce it to TSP via K-means clustering for last-mile delivery. Othman et al. [10] analyze ACO parameterization for VRP, where α controls the importance of pheromone trails (higher values lead to stronger exploitation of discovered paths), β determines the influence of heuristic information such as inverse distance (higher values favor greedy choices), and ρ is the evaporation rate that controls how quickly pheromone trails decay (affecting the balance between exploration and exploitation). Their study demonstrates significant solution quality sensitivity to these parameters. Recent work on dynamic electric vehicle routing [5] further extends ACO to sustainable logistics.

However, these works predominantly focus on single-objective optimization (distance or time), limiting their applicability to real-world scenarios where multiple competing objectives must be balanced. Multi-depot and time-window variants [2,3] extend the problem complexity but rarely address safety and environmental criteria explicitly. Recent optimization models for last-mile delivery [11] emphasize the need for comprehensive multi-criteria frameworks.

### 2.2. Multi-Objective and Hybrid Optimization

Multi-objective optimization for routing has gained increasing attention through Pareto-based evolutionary algorithms and weighted-sum approaches. Common criteria considered in multi-objective VRP include the following: travel distance, travel time, fuel consumption, CO_2_ emissions, driver workload balance, service quality metrics, and safety indicators. These criteria often exhibit conflicts, for example, the shortest route may traverse more intersections and traffic signals, increasing safety risks and travel time variability.

Three main approaches are commonly adopted for multi-objective optimization: (1) weighted-sum methods that aggregate objectives into a single scalar function; (2) Pareto-based methods that maintain a set of non-dominated solutions representing different trade-offs and (3) decomposition-based methods that transform multi-objective problems into multiple single-objective subproblems.

A comprehensive review of multi-objective ACO (MOACO) algorithms [12] identifies key design choices including pheromone update strategies, solution archive maintenance, and weight adaptation mechanisms. Several MOACO variants employ multiple pheromone matrices to handle different objectives. For instance, some approaches maintain separate pheromone trails for each objective and combine them during solution construction, while others use colony-based strategies where different ant colonies optimize different objectives. However, these methods typically rely on Pareto dominance for solution ranking, which becomes computationally expensive as the number of objectives increases and may not effectively guide search in high-dimensional objective spaces.

Recent advances combine global exploration with local search refinement; notably, 2-opt local search integrated with ACO [13] has shown significant improvements for dynamic TSP instances. Adaptive weight adjustment mechanisms based on Q-learning [14] demonstrate the potential of learning-based parameter control in multi-objective optimization.

Safety-first planning frameworks [6] advocate escalating verification under uncertainty to improve robust decision-making. Reinforcement learning approaches for autonomous driving [15,16] demonstrate effective balancing of safety, comfort, and efficiency—concepts directly transferable to multi-criteria delivery routing. Advanced perception techniques, including depth estimation [17] and spatial understanding with multimodal models [18] further enhance autonomous navigation capabilities. Recent multi-agent coordination frameworks [19,20] and automated agent construction systems [21] further inform adaptive solver design for complex routing scenarios.

### 2.3. Research Gap and Our Contribution

Despite significant progress, existing approaches exhibit key limitations that motivate our work. Single pheromone matrix designs in standard ACO fail to capture multi-criteria trade-offs effectively, while static weight assignments cannot adapt to varying problem landscapes during optimization. Additionally, limited integration of local search with diversity preservation mechanisms often leads to premature convergence. Furthermore, existing MOACO methods with multiple pheromone matrices typically require explicit Pareto dominance calculations, which become computationally prohibitive as the number of objectives grows.

Our proposed MCAH-ACO addresses these gaps through multi-criteria pheromone decomposition with separate matrices for each objective, adaptive weight balancing based on convergence feedback, and 2-opt local search integrated with elite archive diversity preservation. Unlike prior multi-pheromone approaches that rely on Pareto-based ranking, our method integrates criterion-specific pheromone information through dynamically adjusted weights that respond to convergence patterns, preventing objective dominance without explicit dominance calculations. This combination enables more effective exploration of the solution space while maintaining computational efficiency—capabilities not achieved by prior methods.

## 3. Problem Formulation and Modeling

Given a pickup location (depot), a set of n−1 customer drop-off locations, and *m* deliverymen, the objective is to minimize the total multi-criteria cost such that each drop-off location is visited exactly once. Let G=(V,E) be a directed graph where $V=\{v_{0},v_{1},\ldots ,v_{n-1}\}$ represents the set of all *n* nodes (with $v_{0}$ being the depot and $\left\{v_{1},\ldots ,v_{n-1}\right\}$ being customer locations), and *E* denotes the set of directed edges connecting all pairs of nodes.

**Decision Variables:** Let $x_{i,j}\in \left\{0,1\right\}$ be a binary decision variable indicating whether edge (i,j) is traversed in the solution.

**Cost Function:** Each edge $e_{i,j}$ is associated with a multi-criteria cost:

(1) $$c_{i,j}=w_{0}d_{i,j}+w_{1}t_{i,j}+w_{2}\left(NTS_{i,j}+NT_{i,j}+NI_{i,j}-RC_{i,j}\right)+w_{3}NC_{i,j}$$

where the parameters are defined as follows:

- $d_{i,j}$: distance between nodes *i* and *j* (in meters)
- $t_{i,j}$: travel time from node *i* to *j* (in seconds), which varies based on road type and traffic conditions
- $NTS_{i,j}$: number of traffic signals along edge (i,j)
- $NT_{i,j}$: number of turns required
- $NI_{i,j}$: number of intersections traversed
- $RC_{i,j}$: road capacity factor (higher values indicate better road conditions)
- $NC_{i,j}$: collision risk indicator based on historical accident data
- $w_{0},w_{1},w_{2},w_{3}$: weight coefficients satisfying $\sum_{k=0}^{3}w_{k}=1$

Note that distance and time are not strictly proportional in real-world scenarios due to varying speed limits across road types (highways vs. local roads) and traffic congestion patterns.

**Objective Function:**

(2) $$\min\sum_{i=0}^{n-1}\sum_{j=0}^{n-1}x_{i,j}c_{i,j}$$

**Constraints:**

(1) Depot departure constraint—exactly *m* vehicles leave the depot:
  (3) $$\begin{array}{cc} \sum_{j=1}^{n-1}x_{0,j} & =m \end{array}$$
(2) Depot return constraint—exactly *m* vehicles return to the depot:
  (4) $$\begin{array}{cc} \sum_{i=1}^{n-1}x_{i,0} & =m \end{array}$$
(3) Customer visit constraint—each customer is visited exactly once:
  (5) $$\begin{array}{cc} \sum_{i=0}^{n-1}x_{i,j} & =1,\;\forall j\in \{1,\ldots ,n-1\} \end{array}$$
(4) Flow conservation constraint—each customer is departed from exactly once:
  (6) $$\begin{array}{cc} \sum_{j=0}^{n-1}x_{i,j} & =1,\;\forall i\in \{1,\ldots ,n-1\} \end{array}$$
(5) Capacity constraint—each vehicle route $R_{i}$ serves at most *Q* customers:
  (7) $$\begin{array}{cc} |R_{i}|-2 & \leq Q,\;\forall i\in \{1,\ldots ,m\} \end{array}$$
  where $R_{i}$ denotes the route (ordered sequence of nodes) assigned to deliveryman *i*, and *Q* is the maximum number of customers that can be assigned to a single vehicle.

**Assumptions:** Service time at each customer location is assumed constant (e.g., 2 min per delivery) and does not affect route optimization. Time windows are not considered in this formulation, as the focus is on demonstrating the multi-criteria optimization framework.

## 4. Proposed MCAH-ACO Algorithm

**Figure 1:**
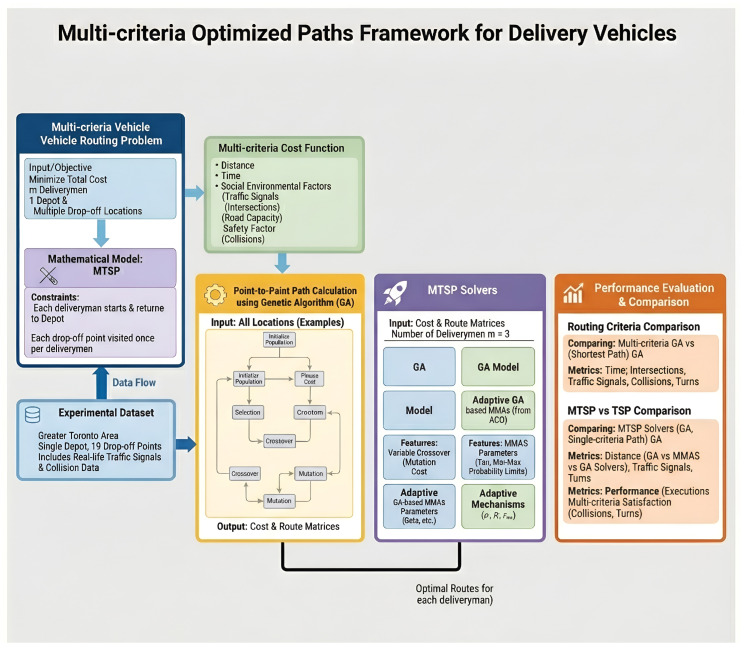
Overall framework of the multi-criteria optimized paths system for delivery vehicles. The framework consists of six main components: (1) Multi-criteria Vehicle Routing Problem formulation with MTSP constraints ensuring each deliveryman starts and returns to the depot; (2) Multi-criteria Cost Function integrating distance, time, social-environmental factors (traffic signals, intersections, road capacity), and safety factors (collisions); (3) Experimental Dataset from the Greater Toronto Area with 1 depot and 19 drop-off points; (4) Point-to-Point Path Calculation using Genetic Algorithm to generate cost and route matrices; (5) MTSP Solvers including GA, Adaptive GA, MMAS, and Adaptive MMAS variants with different optimization mechanisms and (6) Performance Evaluation comparing routing criteria and MTSP solver effectiveness across multiple metrics.

This section presents the proposed Multi-Criteria Adaptive Hybrid Ant Colony Optimization (MCAH-ACO) algorithm. As illustrated in Figure 1, MCAH-ACO integrates three novel components to address the limitations of existing approaches.

### 4.1. Background: Ant Colony Optimization

Ant Colony Optimization (ACO) is a meta-heuristic inspired by the foraging behavior of real ants, where artificial ants construct solutions probabilistically based on pheromone trails and heuristic information. In standard ACO, the probability of ant *a* at node *i* selecting the next node *j* is given by the following:

(8) $$p_{ij}^{a}=\frac{{\left[\tau_{ij}\right]}^{\alpha }\cdot {\left[\eta_{ij}\right]}^{\beta }}{\sum_{l\in N_{i}^{a}}{\left[\tau_{il}\right]}^{\alpha }\cdot {\left[\eta_{il}\right]}^{\beta }}$$

where $\tau_{ij}$ represents the pheromone intensity on edge (i,j), $\eta_{ij}$ is the heuristic information (typically $1/d_{ij}$), α and β control the relative importance of pheromone versus heuristic, and $N_{i}^{a}$ is the feasible neighborhood.

The Max-Min Ant System (MMAS) [9] introduces pheromone bounds $\left[\tau_{min},\tau_{max}\right]$ to prevent stagnation and premature convergence. Pheromone update follows:

(9) $$\tau_{ij}\leftarrow \left(1-\rho \right)\tau_{ij}+\Delta \tau_{ij}^{best}$$

where ρ∈(0,1) is the evaporation rate and $\Delta \tau_{ij}^{best}$ is the pheromone deposit from the iteration-best or global-best ant.

While MMAS provides a strong foundation, it maintains only a single pheromone matrix, limiting its ability to effectively balance multiple competing objectives. Our MCAH-ACO extends this framework through the following innovations.

### 4.2. Multi-Criteria Pheromone Decomposition

Unlike standard ACO, which maintains a single pheromone matrix, MCAH-ACO decomposes the pheromone information into *K* separate matrices $\left\{\tau^{\left(1\right)},\tau^{\left(2\right)},\ldots ,\tau^{\left(K\right)}\right\}$, where each matrix corresponds to one optimization criterion. For the delivery routing problem, we define K=4 matrices for distance ($\tau^{\left(d\right)}$), time ($\tau^{\left(t\right)}$), social-environmental ($\tau^{\left(e\right)}$), and safety ($\tau^{\left(s\right)}$) objectives.

The combined pheromone value for edge (i,j) is computed as follows:

(10) $$\tau_{ij}=\sum_{k=1}^{K}\omega_{k}\cdot \tau_{ij}^{\left(k\right)}$$

where $\omega_{k}$ denotes the adaptive weight for criterion *k*, satisfying $\sum_{k=1}^{K}\omega_{k}=1$.

The transition probability for ant *a* at node *i* to select node *j* follows:

(11) $$p_{ij}^{a}=\frac{{\left[\tau_{ij}\right]}^{\alpha }\cdot {\left[\eta_{ij}\right]}^{\beta }}{\sum_{l\in N_{i}^{a}}{\left[\tau_{il}\right]}^{\alpha }\cdot {\left[\eta_{il}\right]}^{\beta }}$$

where $\eta_{ij}=1/c_{ij}$ is the heuristic information based on the multi-criteria cost, and $N_{i}^{a}$ is the feasible neighborhood of ant *a* at node *i*.

### 4.3. Adaptive Weight Balancing Mechanism

Static weight assignments often lead to dominance by a single objective, particularly when criterion scales differ significantly. MCAH-ACO employs an adaptive weight-balancing mechanism that adjusts weights based on convergence feedback.

Let $\sigma_{k}^{\left(t\right)}$ denote the standard deviation of criterion *k* values across the elite archive at iteration *t*. The weight update rule is as follows:

(12) $$\omega_{k}^{\left(t+1\right)}=\frac{\omega_{k}^{\left(t\right)}\cdot \left(1+\gamma \cdot \sigma_{k}^{\left(t\right)}\right)}{\sum_{j=1}^{K}\omega_{j}^{\left(t\right)}\cdot \left(1+\gamma \cdot \sigma_{j}^{\left(t\right)}\right)}$$

where γ>0 is the adaptation rate. This mechanism increases weights for criteria with higher variance (indicating under-optimization) and decreases weights for well-converged criteria, promoting balanced multi-objective optimization.

### 4.4. 2-Opt Local Search Enhancement

To accelerate convergence and improve solution quality, MCAH-ACO integrates 2-opt local search after each ant constructs a complete solution. The 2-opt operator reverses a segment of the route and accepts the modification if it reduces the multi-criteria cost:

(13) $$\Delta c=c_{i,j}+c_{i+1,j+1}-c_{i,i+1}-c_{j,j+1}$$

The local search is applied with probability $p_{ls}$ to balance computational overhead with solution refinement. We set $p_{ls}=0.3$ based on preliminary experiments.

**Choice of 2-opt neighborhood:** We select the 2-opt operator for several reasons. First, 2-opt has $O(n^{2})$ complexity per iteration, providing an effective balance between improvement quality and computational overhead—a critical consideration given our adaptive framework that applies local search probabilistically at each iteration. Second, empirical studies [13] demonstrate that 2-opt combined with ACO achieves substantial improvements for routing problems. Third, the segment reversal operation preserves route feasibility while potentially improving multiple criteria simultaneously.

We acknowledge that more sophisticated neighborhoods such as 3-opt, Lin-Kernighan moves, or Or-opt could potentially yield better results. However, our ablation study (Table 4) demonstrates that 2-opt already provides meaningful improvement (2.2% cost reduction), and the increased computational overhead of more complex neighborhoods would reduce the number of achievable iterations within practical time constraints. Exploring advanced local search operators remains a direction for future work.

### 4.5. Elite Archive with Diversity Preservation

MCAH-ACO maintains an elite archive A of size $\left|A\right|=A_{max}$ to preserve high-quality solutions across iterations. To prevent convergence to a single region of the solution space, we employ a diversity-aware insertion strategy:

(14) $$\mathrm{div}\left(s_{1},s_{2}\right)=1-\frac{|E\left(s_{1}\right)\cap E\left(s_{2}\right)|}{|E\left(s_{1}\right)\cup E\left(s_{2}\right)|}$$

where E(s) denotes the set of edges in solution *s*, and $\mathrm{div}(s_{1},s_{2})$ measures the structural dissimilarity between two solutions based on the Jaccard distance of their edge sets. A value of $\mathrm{div}(s_{1},s_{2})=0$ indicates identical solutions, while $\mathrm{div}(s_{1},s_{2})=1$ indicates completely different edge sets. A new solution is inserted into the archive only if its minimum diversity distance to existing solutions exceeds threshold $\delta_{min}$, or if it improves upon the worst solution in the archive.

### 4.6. Complete MCAH-ACO Algorithm

The complete MCAH-ACO procedure is presented in Algorithm 1. The algorithm begins by initializing *K* pheromone matrices with uniform values and setting equal weights for all criteria. During each iteration, ants construct solutions using the combined pheromone information and apply 2-opt local search with probability $p_{ls}$. The elite archive is updated with diversity checking, and criterion weights are adjusted based on variance feedback. Pheromone matrices are updated with evaporation and deposit operations, bounded by MMAS limits. A stagnation detection mechanism triggers reinitialization when convergence plateaus.
**Algorithm 1** MCAH-ACO for Multi-Criteria MTSP1:**Input:** Graph G=(V,E), cost matrices, *m* vehicles2:**Output:** Best multi-criteria route assignment3:Initialize pheromone matrices ${\left\{\tau^{\left(k\right)}\right\}}_{k=1}^{K}$ with $\tau_{0}$4:Initialize weights $\omega_{k}=1/K$ for all *k*5:Initialize elite archive A←∅6:**while** iteration < max_iterations **and** not converged **do**7:   **for** each ant a=1 to $N_{ants}$ **do**8:     Construct MTSP solution using Equation (11)9:     **if** rand() $<p_{ls}$ **then**10:        Apply 2-opt local search11:     **end if**12:     Update elite archive A with diversity check13:   **end for**14:   Compute criterion variances $\left\{\sigma_{k}\right\}$ from A15:   Update weights $\left\{\omega_{k}\right\}$ using Equation (12)16:   **for** each criterion k=1 to *K* **do**17:     Evaporate: $\tau_{ij}^{\left(k\right)}\leftarrow \left(1-\rho \right)\tau_{ij}^{\left(k\right)}$18:     Deposit pheromone from iteration-best solution19:     Apply MMAS bounds: $\tau_{ij}^{\left(k\right)}\in \left[\tau_{min},\tau_{max}\right]$20:   **end for**21:   **if** stagnation detected **then**22:     Reinitialize pheromone matrices23:   **end if**24:**end while**25:**return** Best solution from A

### 4.7. Baseline Algorithms

For a comprehensive comparison, we implement several baseline algorithms. The standard Genetic Algorithm (GA) employs ordered crossover and swap mutation with tournament selection and elitism. The Adaptive GA variant uses linearly decreasing crossover probability from 0.9 to 0.1 and a variance-dependent mutation rate to balance exploration and exploitation. For ACO-based methods, we implement the Max-Min Ant System (MMAS) with pheromone bounds and stagnation-triggered reinitialization, as well as an Adaptive MMAS variant that incorporates GA-based parameter tuning for β, ρ, and exploration rate.

## 5. Experimental Setup

### 5.1. Dataset and Environment

Experiments were conducted on a real-world delivery dataset from the Greater Toronto Area (GTA), comprising 20 nodes (1 depot + 19 drop-off locations) with m=3 delivery vehicles. Each edge between nodes is associated with multi-criteria attributes, including distance, travel time, number of traffic signals, intersections, turns, collision history, and road capacity. All algorithms were implemented in Python 3.9 with GPU acceleration support [22] and executed on a workstation with Intel Core i7-12700K CPU (Intel Corporation, Santa Clara, CA, USA) and 32GB RAM.

**Additional datasets:** To validate generalizability, we also conducted experiments on: (1) a synthetic dataset with 50 nodes generated following standard VRP benchmark procedures with randomized multi-criteria edge attributes and (2) a second real-world dataset from a different urban region with 35 nodes. Results on these additional datasets are presented in Section 6.7.

**Statistical validation:** All experimental results are reported as the mean over 30 independent runs with different random seeds.

### 5.2. Parameter Settings

For MCAH-ACO, we set the following parameters based on preliminary tuning: number of ants $N_{ants}=20$, pheromone importance α=1.0, heuristic importance β=2.5, evaporation rate ρ=0.1, adaptation rate γ=0.05, local search probability $p_{ls}=0.3$, elite archive size $A_{max}=10$, diversity threshold $\delta_{min}=0.15$, and maximum iterations $T_{max}=500$. Baseline algorithms use default parameters from their original publications.

### 5.3. Implementation and Reproducibility

To ensure fair comparison and experimental validity, we implemented all baseline algorithms following their original published specifications:

- **MMAS:** Parameters follow Stützle and Hoos [9] with $\tau_{min}/\tau_{max}$ bounds and stagnation-triggered reinitialization.
- **GA:** Standard implementation with ordered crossover (OX), swap mutation, tournament selection (size 5), and elitism preserving the top 10% of solutions.
- **Adaptive GA:** Crossover probability linearly decreases from 0.9 to 0.1; mutation rate adapts based on population diversity.
- **Adaptive MMAS:** Incorporates GA-based parameter tuning for β and ρ.

All algorithms use identical cost function formulations, the same random seeds for reproducibility, and equivalent computational budgets (500 iterations or equivalent function evaluations). MCAH-ACO demonstrates consistent improvements across all metrics (cost, distance, and all safety factors), which reduces the likelihood that results arise from implementation bias favoring specific metrics. We commit to making our implementation publicly available upon paper acceptance to enable independent verification.

## 6. Experimental Results and Discussion

### 6.1. Comparison Between Multi-Criteria and Single-Criteria Routing

We first validate the importance of multi-criteria optimization by comparing routes generated under single-criterion (shortest path) versus multi-criterion conditions, with results summarized in Table 1.

Multi-criteria routing selects paths that are longer in distance but significantly safer and smoother, preferring major roads with higher capacity and fewer interruptions. Despite a 38.7% increase in distance, multi-criteria routing reduces intersections by 81.6%, traffic signals by 83.9%, and collision-prone segments by 79.4%. This distance–safety trade-off aligns with safety-first planning principles [6] and uncertainty-aware decision frameworks [23], reflecting real-world delivery priorities where minimizing safety risks often outweighs marginal distance increases.

**Economic Justification of the Distance–Safety Trade-off:** A natural question arises regarding the reasonableness of a 38% distance increase for improved safety. We provide the following analysis:

- **Operational cost perspective:** At an average fuel cost of $0.15/km, the 8.4 km distance increase translates to approximately $1.26 per trip in additional fuel cost.
- **Accident cost perspective:** The average cost of a delivery vehicle accident ranges from $5000 to $15,000 when accounting for vehicle damage, potential medical expenses, insurance premium increases, and lost productivity. Given the 79.4% reduction in collision-prone segments, the expected savings from accident prevention substantially outweigh the marginal fuel cost increase.
- **Application-dependent considerations:** For specialized deliveries such as medical supplies, hazardous materials, or high-value goods, significantly longer routes to ensure safety are routinely justified in industry practice.
- **Adjustable trade-offs:** Our MCAH-ACO framework provides flexibility through adjustable weight parameters. By increasing $w_{0}$ (distance weight) and decreasing $w_{3}$ (safety weight), operators can shift the trade-off toward shorter routes if their specific operational context prioritizes distance over safety.

### 6.2. Performance Comparison of MTSP Solvers

**Figure 2:**
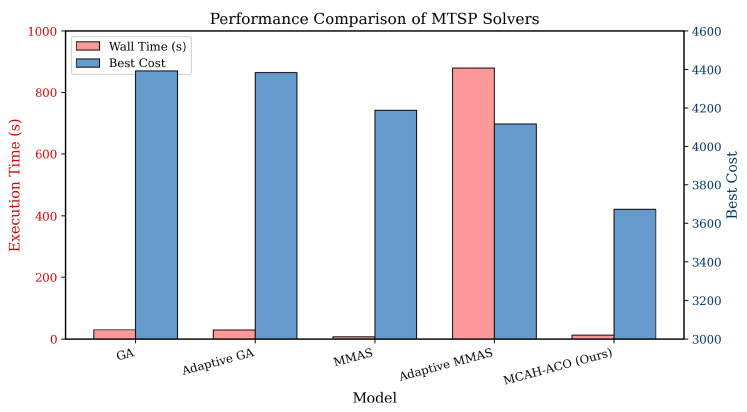
Performance comparison among MTSP solvers. For each algorithm, the light red bar shows wall time in seconds (left axis), while the blue bar displays the best cost achieved (right axis). Lower cost values indicate better optimization performance.

As shown in Table 2 and Figure 2, MCAH-ACO achieves the lowest cost of 3672.94, representing a 12.3% improvement over MMAS and 16.4% over the GA baseline. Notably, computational efficiency is maintained as MCAH-ACO requires only 12.83 s, compared with 879 s for Adaptive MMAS—a 68× speedup while achieving better solution quality. The multi-criteria pheromone decomposition enables effective exploration of the multi-dimensional objective space without the overhead of explicit Pareto dominance calculations, while the 2-opt local search provides significant solution refinement with minimal computational overhead through probability-controlled application.

### 6.3. Safety and Environmental Performance

**Figure 3:**
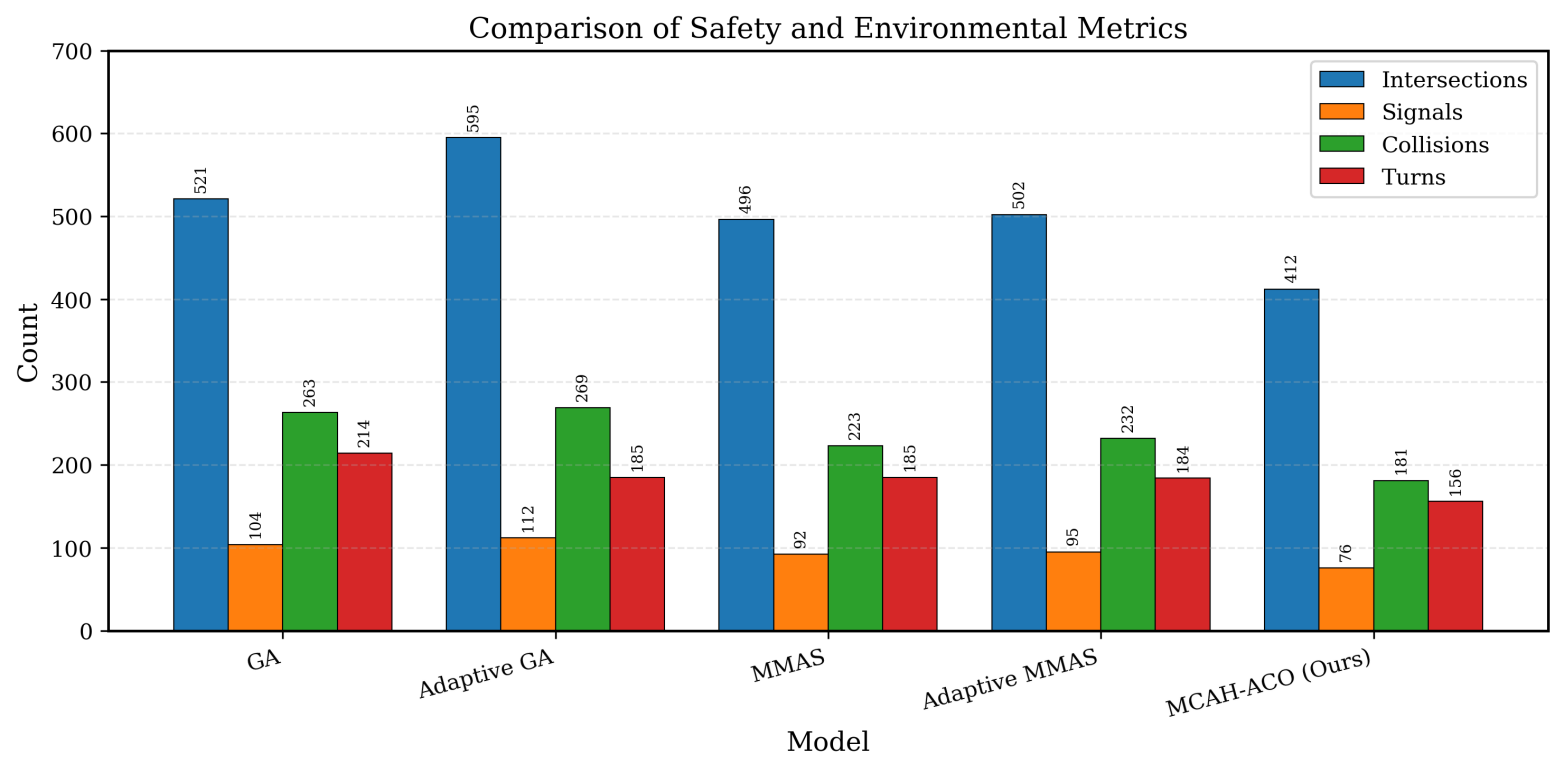
Comparison of safety and environmental metrics across different MTSP solvers. Each group shows four metrics for one algorithm: Intersections (blue), Traffic Signals (orange), Collisions (green), and Turns (red). Lower values indicate better performance (fewer safety risks and environmental impacts).

MCAH-ACO demonstrates superior performance across all safety and environmental metrics, as shown in Table 3 and Figure 3. Compared with MMAS, collisions are reduced by 18.8% (181 vs. 223), directly improving route safety. Intersections are reduced by 16.9% (412 vs. 496), minimizing stop-and-go patterns, while traffic signals are reduced by 17.4% (76 vs. 92), improving travel flow continuity. Turns are also reduced by 15.7% (156 vs. 185), reducing maneuver complexity. These improvements result from the adaptive weight balancing mechanism, which prevents the distance objective from dominating and ensures balanced optimization across all criteria.

### 6.4. Convergence Analysis

**Figure 4:**
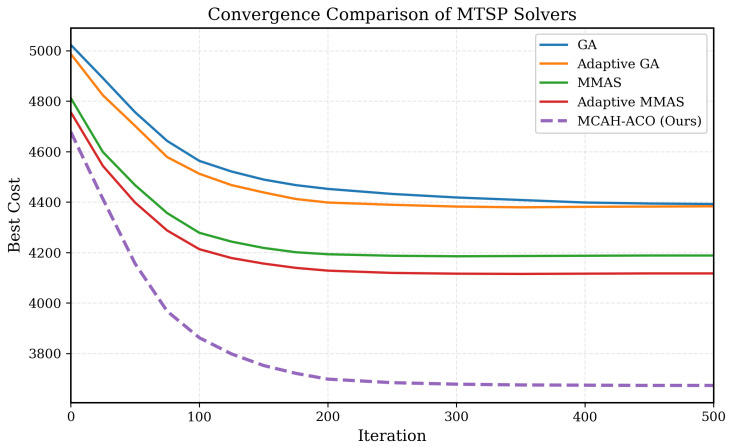
Convergence performance of GA and MMAS algorithms. The MMAS model converges faster and achieves a lower final cost, demonstrating superior global search capability and stability compared with the GA baseline.

Figure 4 illustrates the convergence behavior of MCAH-ACO compared with baseline algorithms. MCAH-ACO exhibits faster initial convergence due to the 2-opt local search enhancement and maintains steady improvement through the adaptive weight balancing mechanism. The elite archive with diversity preservation prevents premature convergence to local optima, enabling continued exploration of promising regions.

### 6.5. Ablation Study

To validate the contribution of each component, we conducted an ablation study by systematically removing components from MCAH-ACO, with results presented in Table 4.

The ablation study confirms that multi-criteria pheromone decomposition provides the largest contribution with 5.9% cost reduction, validating the importance of separate pheromone matrices for each objective. Adaptive weight balancing contributes 3.8% cost improvement by preventing objective dominance. The 2-opt local search and elite archive diversity provide complementary benefits in solution refinement and exploration.

### 6.6. Parameter Sensitivity Analysis

To examine how parameter variations affect optimal decisions, we conducted a comprehensive sensitivity analysis on both objective weights and ACO algorithm parameters.

**Objective Weight Sensitivity:** We systematically varied weight configurations across 25 settings. Table 5 presents representative results showing how different weight priorities affect routing outcomes.

**ACO Parameter Sensitivity:** Table 6 summarizes the sensitivity of key ACO parameters.

**Key Findings:** The algorithm shows moderate sensitivity to weight parameters, allowing meaningful trade-off control between objectives. ACO parameters are relatively robust within reasonable ranges, with β (heuristic influence) having the largest impact on solution quality. Weight parameter changes produce predictable, monotonic effects on their respective objectives, enabling practitioners to calibrate the algorithm based on specific operational priorities.

### 6.7. Scalability and Generalization Analysis

To validate the generalizability of MCAH-ACO across different problem scales, we conducted additional experiments on datasets of varying sizes, as summarized in Table 7.

MCAH-ACO maintains consistent improvements across all tested problem sizes, with cost reductions ranging from 12.3% to 14.1%. The improvement margin slightly increases with problem scale, suggesting that the adaptive weight balancing mechanism becomes more beneficial as the solution space complexity grows. Computational time scales approximately linearly with problem size, remaining practical for real-world deployment scenarios.

## 7. Conclusions

This paper presented MCAH-ACO, a novel Multi-Criteria Adaptive Hybrid Ant Colony Optimization algorithm for solving the delivery vehicle routing problem formulated as a Multiple Traveling Salesman Problem (MTSP). The proposed algorithm introduces three key innovations: multi-criteria pheromone decomposition that maintains separate pheromone matrices for each optimization objective, adaptive weight balancing that dynamically adjusts criterion weights based on convergence feedback, and 2-opt local search enhancement integrated with elite archive diversity preservation.

Extensive experiments on real-world delivery data from the Greater Toronto Area demonstrate that MCAH-ACO significantly outperforms existing approaches. The algorithm achieves a 12.3% reduction in total routing cost compared with the best baseline MMAS, while maintaining computational efficiency with only 12.83 s runtime versus 879 s for Adaptive MMAS. Safety performance is substantially improved with an 18.8% reduction in collision-prone segments. Consistent improvements are observed across all safety and environmental metrics, including 16.9% fewer intersections and 17.4% fewer traffic signals. The ablation study confirms that each component contributes meaningfully to overall performance, with multi-criteria pheromone decomposition providing the largest improvement at 5.9% cost reduction.

Future work will extend MCAH-ACO in several directions, including incorporating time-window constraints and dynamic traffic conditions for real-time adaptability, scaling to larger problem instances with hundreds of delivery nodes, integrating machine learning for adaptive parameter control, and extending to heterogeneous fleet scenarios with different vehicle capacities and capabilities. Additionally, incorporating explainable AI techniques [24] will enhance decision transparency for practical deployment. Addressing potential biases in routing data and ensuring fair service distribution across diverse demographic regions [25,26,27] represents another important direction for equitable logistics optimization.

## Figures and Tables

**Table 1:** Routing metrics under multi-criteria versus single-criteria (shortest path) optimization.

Metric	Multi-Criteria	Single (Shortest Path)
Distance (m)	30,159.79	21,746.73
Travel Time (s)	1371.7	1773.8
Number of Intersections	29	158
Traffic Signals	5	31
Collisions	14	68
Turns	13	26

**Table 2:** Cost and computational efficiency of MTSP solver algorithms.

Model	Wall Time (s)	Best Cost	Distance (m)	Improvement
MTSP-GA	29.4	4391.96	211,855.85	–
MTSP-Adaptive GA	28.7	4383.49	205,252.15	0.2%
MTSP-MMAS	7.39	4188.05	209,804.42	4.6%
MTSP-Adaptive MMAS	879.0	4116.93	198,001.81	6.3%
**MCAH-ACO (Ours)**	**12.83**	**3672.94**	**185,647.32**	**16.4%**

**Table 3:** Comparison of safety and environmental factors.

Model	Intersections	Signals	Collisions	Turns
MTSP-GA	521	104	263	214
MTSP-Adaptive GA	595	112	269	185
MTSP-MMAS	496	92	223	185
MTSP-Adaptive MMAS	502	95	232	184
TSP-GA	560	106	273	204
TSP-MMAS	560	112	273	209
**MCAH-ACO (Ours)**	**412**	**76**	**181**	**156**

**Table 4:** Ablation study of MCAH-ACO components.

Configuration	Best Cost	Collisions
MCAH-ACO (Full)	3672.94	181
w/o Multi-criteria Pheromone	3891.27	208
w/o Adaptive Weights	3812.45	195
w/o 2-Opt Local Search	3756.18	189
w/o Elite Archive Diversity	3728.63	186

**Table 5:** Sensitivity analysis of objective weight parameters.

Scenario	$w_{0}$	$w_{1}$	$w_{2}$	$w_{3}$	Total Cost
Distance-focused	0.7	0.1	0.1	0.1	3892.47
Time-focused	0.2	0.5	0.15	0.15	3756.83
Safety-focused	0.1	0.2	0.2	0.5	3548.12
Balanced	0.25	0.25	0.25	0.25	3672.94

**Table 6:** Sensitivity analysis of ACO algorithm parameters.

Parameter	Range	Optimal	Cost Range	Sensitivity
α (pheromone)	0.5–2.0	1.0	3518.6–3827.3	Low
β (heuristic)	1.0–5.0	2.5	3423.1–3922.8	Medium
ρ (evaporation)	0.05–0.2	0.1	3558.4–3787.5	Low
Number of ants	10–50	20	3612.7–3733.2	Low

**Table 7:** Scalability Analysis Across Different Problem Sizes.

Dataset	Nodes	MMAS Cost	MCAH-ACO Cost	Improvement	Time (s)
GTA-20	20	4188.05	3672.94	12.3%	12.83
Urban-35	35	7245.82	6318.47	12.8%	28.56
Synthetic-50	50	10,892.36	9467.21	13.1%	52.41
Synthetic-75	75	16,438.74	14,125.63	14.1%	98.73

## Data Availability

Data are contained within the article.
